# Expanded Neonatal Bloodspot Screening Programmes: An Evaluation Framework to Discuss New Conditions With Stakeholders

**DOI:** 10.3389/fped.2021.635353

**Published:** 2021-02-22

**Authors:** Marleen E. Jansen, Anne W. Klein, Erika C. Buitenhuis, Wendy Rodenburg, Martina C. Cornel

**Affiliations:** ^1^Centre for Health Protection, National Institute for Public Health and the Environment, Utrecht, Netherlands; ^2^Department of Clinical Genetics, Section Community Genetics and Amsterdam Public Health Research Institute, Amsterdam University Medical Centers, Vrije Universiteit Amsterdam, Amsterdam, Netherlands; ^3^Centre for Population Screening, National Institute of Public Health and the Environment, Utrecht, Netherlands

**Keywords:** neonatal screening, implementation, stakeholders, decision making, public health policy cycle, evaluation framework

## Abstract

Neonatal bloodspot screening (NBS) programmes that screen for rare but serious conditions are expanding worldwide. Fast developments for testing and treatment put pressure on implementation processes. In 2015 the Netherlands embarked on an NBS expansion from 17 to 31 conditions. An evaluation framework was developed based on international NBS frameworks to gain insight in test properties, clinical findings, follow-up and implementation. A stakeholder process took place with implications for the planning of the expanded NBS panel. The evaluation framework progressed into a *go/no go* framework to start national screening, and is currently explored as basis for continuous evaluation of the NBS panel. The framework and stakeholder process may serve as an example for other programmes.

## Introduction

Neonatal bloodspot screening (NBS) programmes are important and successful public health initiatives for early recognition of rare, congenital disorders (1). NBS usually focuses on disorders for which early detection enables early intervention that prevents or minimises irreversible health damage. In the Netherlands newborns are screened for 24 conditions as of January 2021 (Table 1). NBS can only reach its potential to prevent health damage in babies when the programmes are effectively organised. As we will show in this policy brief, NBS programmes are complex (3), and all steps of the public health policy cycle need to be carefully considered (4, 5): agenda setting; policy advice; policy decision; implementation; and evaluation.

**Table 1:** Neonatal screening programme in the Netherlands summarised.

**Screening process**	**Conditions in the current programme (1 February 2021)**	**Conditions included in the expansion**
All new Dutch parents are offered NBS for their child. Annually, over 99% of ~170,000 Dutch neonates undergo NBS (2). A few drops of blood are obtained from the heel and collected on a filter paper card between 72 and 168 h post-partum. The cards with the dried bloodspots are sent to one of five Dutch regional screening laboratories and analysed. After analyses, the cards are pseudonymised through an encrypted barcode and stored centrally at the national reference laboratory. The national NBS program has been expanded from 17 to 24 conditions since January 2021. It is expected to be expanded with 8 additional conditions in the coming years.	1. Alpha-thalassemia (HbH-disease) 2. Beta thalassemia major (TM) 3. Biotinidase deficiency (BIO) 4. Carnitine palmitoyltransferase deficiency type 1 (CPT1) 5. Congenital adrenal hyperplasia (CAH) 6. Congenital hypothyroidism (CH) 7. Cystic fibrosis (CF) 8. Galactosemia (GAL) 9. Galactokinase deficiency (GALK) 10. Glutaric acidemia type I (GA-1) 11. HMG-CoA-lyase deficiency (HMG) 12. Isovaleric acidemia (IVA) 13. Long-chain hydroxyacyl-CoA dehydrogenase deficiency (LCHADD) 14. Multiple CoA Carboxylase deficiency (MCD) 15. Maple syrup urine disease (MSUD) 16. Medium-chain acyl CoA dehydrogenase deficiency (MCADD) 17. 3-Methylcrotonyl-CoA carboxylase deficiency (3-MCC) 18. Methylmalonic acidemia (MMA) 19. Phenylketonuria (PKU) 20. Propionic acidemia (PA) 21. Severe combined immune deficiency (SCID) 22. Sickle cell disease (SCD) 23. Type 1 tyrosinemia (TYR-1) 24. Very long-chain acylCoA dehydrogenase deficiency (VLCADD)	25. Carnitine-acylcarnitine translocase deficiency (CACT) 26. Carnitine palmitoyltransferase deficiency type 2 (CPT2) 27. Guanidinoacetate methyltransferase deficiency (GAMT) 28. Methyl-acetoacetyl-CoA thiolase deficiency, ketothiolase deficiency (BKT) 29. Mucopolysaccharidosis type 1 (MPS I) 30. Organic cation transporter 2 deficiency (OCTN 2) 31. Spinal muscular atrophy (SMA)^a^ 32. X-linked adrenoleukodystrophy (ALD)

*NBS, neonatal bloodspot screening; RIVM, National Institute for Public Health and the Environment*.

a *Addition of SMA was advised and decided on in 2019–2020*.

Since its initiation in the 1960s with screening for phenylketonuria (PKU), innovations have accelerated expansion of NBS programmes. An archetypical example is the introduction of tandem mass spectrometry (MS/MS), which facilitated simultaneous biochemical analyses for a significant number of metabolic disorders. The availability of MS/MS led to test-driven expansions in NBS programmes worldwide, resulting in some programmes currently screening more than fifty conditions (1, 6). Innovations both in treatments and test methods continue, enabling screening for additional conditions. When an intervention with substantial health benefit is proven effective for a neonatal condition and a suitable screening test is available, the condition becomes eligible for screening. A combination of a suitable test and effective treatment have recently made severe combined immunodeficiency (SCID) and spinal muscular atrophy (SMA) eligible for screening, and they are gradually implemented in NBS programmes.

While significant treatment- and test-driven expansions are seen in several NBS programmes worldwide, other NBS programmes expand at a slower rate (7). This illustrates that even though screening tests and treatments are available, the local context will determine the NBS program put in place (5, 8). Differences in local context include for example available resources, disease prevalence, and (interpretation of) screening criteria. For the latter, the Wilson and Jungner screening principles are used since their publication in 1968 and revision in 2008 (9, 10). However, heterogeneity in applying these principles for decision-making contributes to the different NBS programmes we see today. For example, the Health Council of the Netherlands (GR) translated the 10 principles into 12 criteria, not only focusing on the question whether a screening should be introduced, but also how. It included elements on practical aspects, such as quality control after a screening is introduced, but also other important aspects, such as cost-effectiveness (11, 12).

To structure the implementation of fourteen new conditions to the Dutch NBS program, an evaluation framework was developed based on international NBS frameworks. This evaluation framework focused on the translation from policy to practice. It included aspects for insight on how to implement the expanded screening programme, specifically for test properties, clinical findings, and follow-up. To implement screening for each condition in the expanded NBS programme in a timely but responsible manner, a stakeholder process was employed, which we will summarise here. While the process and framework summarised can be used by public health professionals in other countries as a starting point for the implementation process, local contexts for other countries and for example approaches to agenda setting are not discussed. As NBS programmes are expected to continue to expand, policy processes that support timely and responsible decision-making and implementation are paramount.

## NBS Policy in the Netherlands

Various governmental bodies are involved in the (re)assessment and implementation of NBS. The three main parties that are discussed here are the (1) Ministry of Health, Welfare and Sport (VWS) (2) GR, and (3) Centre for Population Screening of the National Institute for Public Health and the Environment (RIVM-CvB):

1. The Ministry of VWS bears political responsibility for NBS and defines the NBS-policy, including the legal and policy framework. It is also responsible for the programme's funding and facilitation of the co-ordination of the programme, which is delegated to RIVM-CvB.
2. The GR is an independent national scientific advisory body. Pursuant to the Public Health Act, the GR has the task of advising ministers and parliament on public health and research into health and healthcare. Ministers ask the GR for advice to underpin policy decisions.
3. RIVM-CvB co-ordinates the national NBS programme. RIVM-CvB directs NBS and manages the implementation, to ensure that the legal and policy frameworks, the public values and clinical care are aligned.

**Figure 1:**
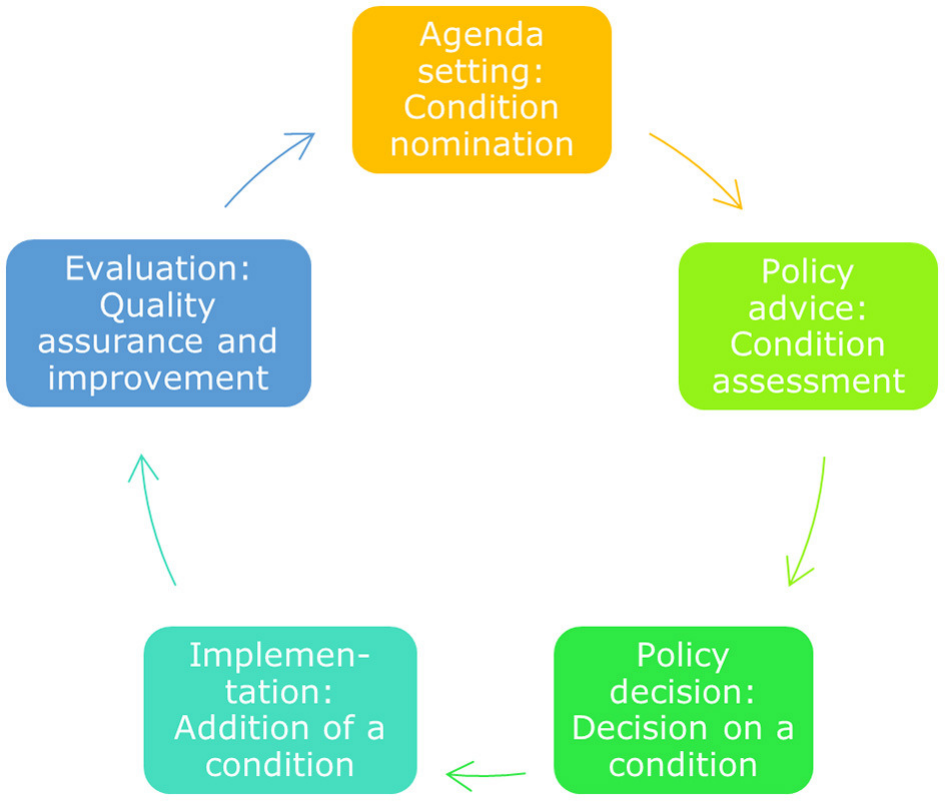
The public health policy cycle. The public health policy cycle consists of five phases. These five phases translate to specific activities for NBS, from nomination of a condition to its quality assurance after implementation (5).

Below we will summarise the policy cycle for public health screening programmes applied to NBS (Figure 1). Then we will report on the policy process that was followed to implement the expanded NBS panel in phases.

### Agenda Setting

In the Netherlands, the agenda for NBS is set on a national level by the Ministry of VWS. When evaluation of the NBS programme as a whole or for a specific condition is on their agenda, the Ministry asks scientific advice from the GR (see Policy Advice).

### Policy Advice

To develop a policy advice, the GR summarises scientific evidence related to the questions from the Ministry. It has a number of permanent committees, which advise on specific areas and take care of consistency in those advices over time. Since 2019 one of these committees is the pre- and neonatal screening committee. Before 2019 NBS was an *ad hoc* committee when evaluation was needed. The advice the GR provides is always based on published, peer-reviewed evidence, reviewed by experts and supplemented with expert opinions when needed.

The GR has advised the Ministry of VWS on NBS expansions upon request in 2005 (advice to add 14 conditions), 2011 (advice to add cystic fibrosis), 2015 (advice to add 14 conditions) and 2019 (advice to add SMA) (12–15). Depending on the GR advice and the Ministry's initial decision, the Ministry requests a feasibility advice from RIVM-CvB for a (group of) condition(s). The advice of RIVM-CvB focusses on the practical feasibility of the programme.

### Policy Decision

Based on the GR and RIVM-CvB advice, respectively through a scientific report (GR) and a feasibility study (RIVM-CvB), the Ministry takes a decision to change the national screening programme or not. In 2015, the GR advised to add fourteen conditions to the NBS programme and in 2017 RIVM-CvB advised a phased implementation of these conditions over a period of 5 years. The Ministry followed this advice, and initiated the implementation phase. This was different from the expansion advised on in 2005 when the Minister of Health, Welfare and Sport (VWS) decided to implement screening on all proposed conditions by 2007. This relatively quick expansion led to some suboptimal results, such as high numbers of false positives.

### Implementation

After the decision of the Ministry of VWS to start implementation, RIVM-CvB embarks on realising the implementation steps evaluated in the feasibility study. RIVM-CvB assures programme quality by setting requirements and monitoring them, such as programme organisation, guidelines, and accreditation requirements. An important advisory committee for RIVM-CvB is the NBS Programme Committee. This committee contains experts from relevant professional and patient organisations. For each condition in the expansion, a final decision to start screening is made by the Ministry, based upon a final advice by the RIVM-CvB. This final decision is based on a positive assessment using the *go/no go* framework, which was developed after the stakeholder process in 2016 and is discussed in the section Stakeholder process. This framework includes all the requirements that have to be in place before starting screening.

### Evaluation

Evaluation of the NBS policy takes place on different levels. Shortly after the introduction of a new condition to the programme, the performance parameters of screening are carefully monitored by RIVM-CvB. RIVM-CvB informs the Ministry of VWS on the outcomes, such as false positives.

RIVM-CvB also alerts and advises the Ministry and other governmental parties about longer term developments and major changes, including innovations, that are important for NBS and that require measures and/or policy changes. The continuous evaluation of the whole NBS programme to complete the policy cycle is carried out on some parameters, but is also under development to monitor an increasingly complex programme through long-term follow-up.

## Stakeholder Process

### Insight in the Practical Feasibility

In 2015 the GR published an advice to expand the NBS programme from 17 to 31 conditions. The Ministry of VWS followed this advice and assigned RIVM-CvB to study the feasibility of adding these fourteen conditions. To initiate the feasibility study, the GR advice was complemented with additional information from grey literature and expert opinion. To structure the additional information, an evaluation framework was developed including aspects, such as Dutch prevalence numbers, test characteristics, and consensus in clinical follow-up in Dutch hospitals.

International policy frameworks were investigated before drawing up the evaluation framework for the feasibility of implementing a new condition. An internet search was conducted and international contacts were approached, resulting in the identification of six frameworks based on previous work (16). These were the frameworks from Australia, Canada (Ontario), Denmark, New Zealand, United Kingdom, and United States of America. The identified frameworks are used for the initiation or cessation of screening, and are therefore very comprehensive.

**Figure 2:**
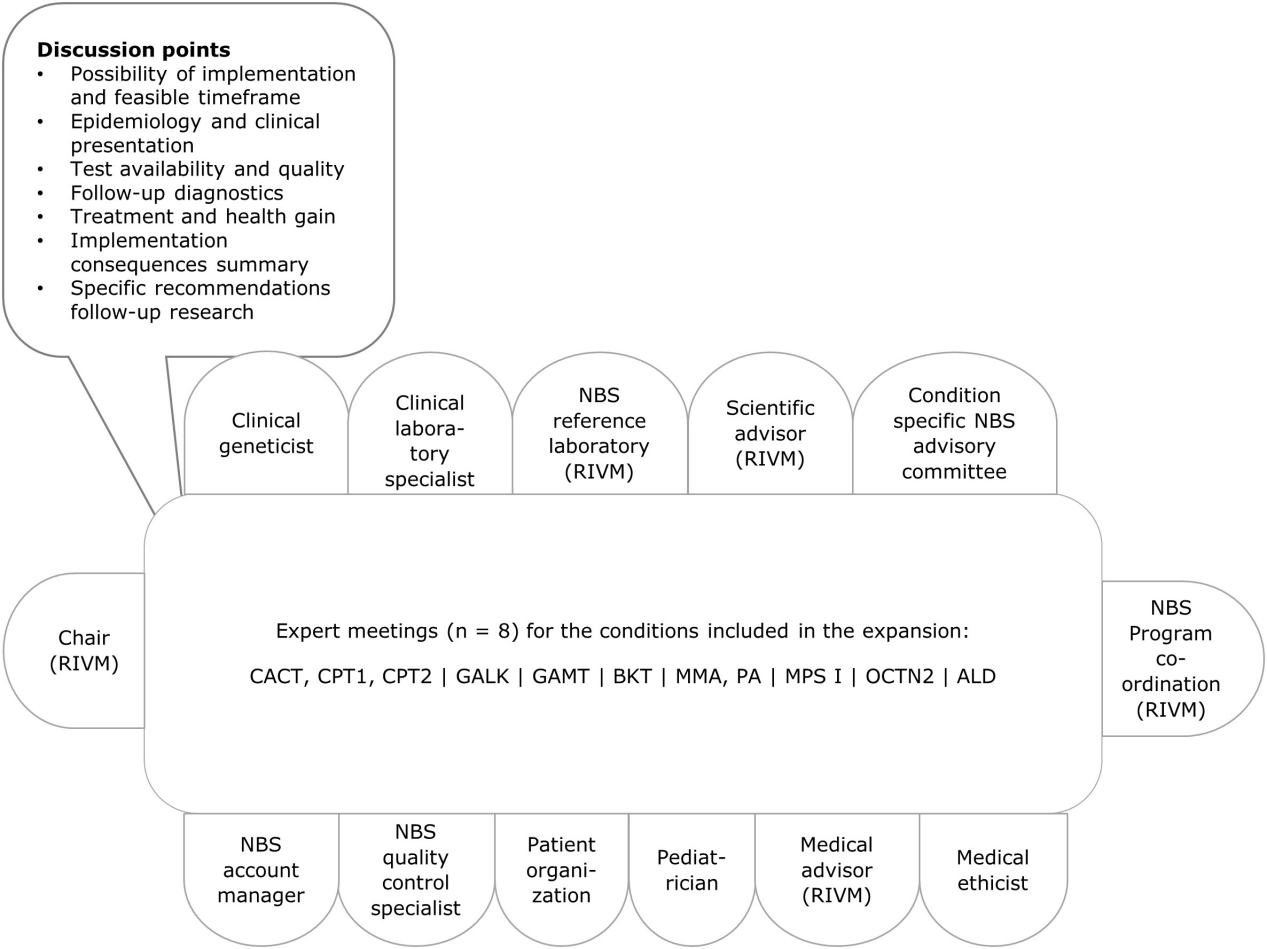
Stakeholders involved and agenda with discussion points. Per condition relevant changes were made to the composition of the group, for example including a metabolic paediatrician or a neuromuscular paediatrician. ALD, X-linked adrenoleukodystrophy; BKT, methyl-acetoacetyl-CoA thiolase deficiency, ketothiolase deficiency; CACT, carnitine-acylcarnitine translocase deficiency; CPT1, carnitine palmitoyltransferase deficiency type 1; CPT2, carnitine palmitoyltransferase deficiency type 2; GALK, galactokinase deficiency; GAMT, guanidinoacetate methyltransferase deficiency; MMA, methylmalonic acidemia; MPS I, mucopolysaccharidosis type 1; PA, propionic acidemia; OCTN2, organic cation transporter 2 deficiency; RIVM, National Institute for Public Health and the Environment.

Since the aim of our evaluation framework was primarily to gain insight into the practical feasibility of adding a condition to the NBS programme, for example cost-effectiveness is not included in the *go/no go* framework, because the GR evaluates this aspect in their role in the decision making process. We focused on characteristics related to the execution of screening, such as availability of a test method in the Netherlands. These aspects are part of the feasibility study by RIVM-CvB. The items were the core of the discussion within the expert groups (Figure 2). We used the evaluation framework to gather information and to structure the discussion, so the expert groups could reach a standardised decision with regard to the (group of) condition(s) to be added to the NBS programme.

### Conducting Expert Group Meetings

RIVM-CvB facilitated a stakeholder process to gain insight in the practical feasibility of screening. First, RIVM established *ad hoc* experts groups for each (group of) condition(s) and organised expert meetings. The expert groups met once for each (group of) condition(s) between June 2016 and October 2016 at RIVM. Prior to all expert meetings an elaborate background document was prepared with information from academic literature, policy reports, conference presentations and information from the project team's (inter)national network. When relevant, experts were asked to prepare a short presentation for the group. Discussion points for the different meetings were based on the information gathered for the evaluation framework (Figure 2).

During each meeting, the experts advised RIVM on potential knowledge gaps and appropriate follow-up (research) to overcome these gaps. They gave an estimation of a feasible timeframe for implementation taking this follow-up (research) into account. For most conditions follow-up research was advised by the experts, mostly validation studies in the Dutch context. For example, studies were proposed to evaluate cut-off values relevant for the Dutch population and screening setting, and clinical outcomes. Furthermore, additional condition-specific research was suggested for X-linked adrenoleukodystrophy (ALD), organic cation transporter 2 deficiency (OCTN-2), and guanidinoacetate methyltransferase deficiency (GAMT). For ALD a pilot study was recommended, since only boys are to be screened which poses significant challenges to the programme (17). For OCTN-2 a study into the predictive value was advised, especially since symptom-free mothers are often detected instead of sick newborns (18). Additional information on the test method was considered necessary for GAMT, as it would be the first fully in-house test method applied in the Netherlands for all neonatal samples.

### Outcomes of the Stakeholder Process

The expert meetings resulted in advisory documents for each condition, with information summarised for each topic from the evaluation framework and suggestions for follow-up (research). Where uncertainties existed, pilot studies and other research projects were advised to resolve the unanswered questions, to be funded by the Organisation for Health Research and Development (ZonMw). The additional information from both the Dutch experts and international experience led to a shift in the previously suggested phasing by the Ministry: three conditions were expected to be implemented sooner, and one later. These outcomes were provided to and discussed with a large group of stakeholders at a meeting held in February 2017. The members of the expert groups, the programme committee, the working group on finance, Youth Health Centre, Dutch Midwives Association, Dutch General Practitioner Association, umbrella association for care organisations, GR and the Ministry of VWS were all invited to this meeting. Finally, the feasibility study for the 14 conditions was compiled and presented to the Ministry of VWS in July 2017.

### From Evaluation to *go/no go* Framework

After the decision of the Ministry of VWS to initiate the implementation of fourteen conditions to the NBS programme, the evaluation framework used in the stakeholder process was developed into a *go/no go* framework for each of the conditions. The combined criteria make a framework which the RIVM-CvB uses to advise the Ministry of VWS on how to add each of the conditions to the programme, and covers topics, such as the test method, the follow-up in healthcare, but also communication and education, costs and legal requirements (Supplementary Table 1). The *go/no go* framework is used to prevent overlooking aspects that are of importance when deciding on the start of screening for a new condition.

A condition-specific implementation plan is based upon the framework, as each criterium is scored green, orange or red to indicate the readiness of implementation on that item (Supplementary Table 1). For each condition the implementation plan is discussed in multidisciplinary project teams, similar to the expert groups during the stakeholder process, and the NBS Programme Committee. The RIVM-CvB provides an advice on the *go* or *no go* for screening to the Ministry of VWS in which input of the discussion with stakeholders is used.

### Using the *go/no go* Framework

The *go/no go* framework has been used for eight conditions (BKT, CACT, CPT1, CPT2, GALK, MMA, MPS I, and PA, Table 1). It was useful to identify and address the critical points for implementation. It appeared to be challenging to decide on cut-off values for the conditions, due to the rare nature of conditions in NBS, and availability of patient samples. Moreover, the clinical follow-up when a condition will be included in NBS is not always straightforward, this can pose a challenge to reach consensus or lead to a “no go” recommendation.

Applying the framework in different countries, could be challenging if the stakeholder network and support from government is different from the Dutch situation. Nonetheless, involving stakeholders as much as possible early on is highly recommended.

## Actionable Recommendations and Conclusions

### Stakeholder Process

Using the knowledge and expertise of the stakeholders in the NBS programme is of crucial importance to the programme. This is true both for a situation in which the programme is expanded with new disorders and in evaluating the existing programme. Bringing the stakeholders together and regularly discussing relevant topics leads to new insights, for example concerning the timing of an addition to the program or appropriate clinical follow-up.

Employing a stakeholder process as part of the policy process provided suggestions for additional research and an updated timeline for the implementation of the expansion of the NBS programme. Furthermore, it facilitated support for the upcoming expansion and offered a network of (new) experts while preparing the implementation. The role of a government agency to facilitate the stakeholder process is important to offer a neutral presentation of perspectives and to include a broad range of stakeholders. Stakeholders to include in the NBS programme are for example patients and patient organisations, parents, paediatricians, internal medicine specialists, laboratory experts, scientific researchers and advisors, clinical geneticists, medical ethicists, and medical advisors.

### Roles and Responsibilities of GR, VWS, RIVM

After a policy advice of the GR, based on peer-reviewed publications and expert opinion, the Minister of VWS decides whether a certain condition should be added to the national NBS programme. Subsequently RIVM investigates how this could be implemented, and which uncertainties should be solved or choices that need to be made before the start of screening. Shortly after the condition has been added to the NBS programme, RIVM monitors and evaluates initial results and whether these meet the expectations. It feeds back the results to GR, VWS, and other stakeholders, both short-term and long-term, to make it possible to complete the policy cycle.

As a lesson learned from the previous expansion (2005–2007), for the ongoing expansion extra focus was put on the practical feasibility of screening for the new conditions, such as the potential number of false positives. During the stakeholder process that was employed for the expansion, the applied evaluation framework proved very useful in structuring the discussion. Furthermore, the stakeholder process inspired another framework to assess the *go/no go* for each condition before the actual implementation. Stepping through the policy cycle together with a broad representation of stakeholders, and using this additional implementation step has facilitated a more robust implementation process.

### Conclusions

Sustainability of NBS programmes is an important topic, as the programmes become increasingly complex due to expansions. To further support the sustainability of NBS programmes, closing the public health policy cycle with evaluation and changes to a programme when needed requires more attention. As the framework presented here is based on international examples, and some of these frameworks are designed to be applied for the initiation or cessation of screening for a condition, large parts of this framework could also be applicable to evaluate a condition that is currently included in a NBS programme. Recently, New Zealand applied their screening framework to evaluate OCTN-2 and decided to discontinue screening for it (18). In the Netherlands, the Ministry of VWS has recently asked the GR to evaluate the current screening panel on its sustainability. The applicability of the *go/no go* framework will also be explored in this evaluation.

Test- and treatment driven expansions of NBS programmes occur globally. After decisions to add a certain condition to a screening programme, the complex aspects of how to implement each step of the screening process for each condition require a systematic and sustainable approach. We suggest a *go/no go* framework used in a stakeholder process with broad representation of NBS stakeholders. Furthermore, the international NBS community could benefit from not only sharing experiences on implementation of conditions, but also on other aspects from the policy cycle, such as agenda setting in collaboration with the local government.

## Author Contributions

MJ contributed to the conception and design of the work, data collection, analysis and interpretation, drafted the work, and revised it. AK contributed to the design of the work, collection and interpretation of data, and critically revised the work. EB contributed to the design of the work and critically revised it. WR contributed to the conception of the work, data collection, and critically revised the work. MC contributed to the conception and design of the work, interpretation of data, and critically revised the work. All authors contributed to the article and approved the submitted version.

## Conflict of Interest

The authors declare that the research was conducted in the absence of any commercial or financial relationships that could be construed as a potential conflict of interest.
