# Supplementary material for: Expanded Neonatal Bloodspot Screening Programmes: An Evaluation Framework to Discuss New Conditions With Stakeholders
Source: Front Pediatr. 2021 Feb 22;9:635353. doi: 10.3389/fped.2021.635353 (PMC7938310; doi:10.3389/fped.2021.635353)
Supplement: Supplementary file 1 [file Table_1.DOCX]

| **Supplementary table 1. *Go / no go* framework**. To visualize the checklist a traffic light method was introduced. If the answer to a question does not hamper implementation, the right column contains a green square . If the answer is not an acute obstruction to proceed with the implementation, but is not according to the initial expectations of the experts and/or further exploration is needed, the right column contains an orange triangle ▲. If the answer makes the expansion of the neonatal heel prick screening test with this condition impossible (for now), the right column contains a red circle ●. |
| --- |
| - Condition (clearly defined, prevalence, incidental findings, mild variants, genetic carriers). - Method of testing (number of tiers, test possible on dried bloodspots, amount of blood needed, analytes, cut-off points, post-analytical tool needed?, laboratory equipment and test kits, quality standards, interference of test with test on other conditions?, demands on logistics and personnel of laboratory). - Predictive value (true positive, false positive, false negative) acceptable? - Time of heel prick screening - Transfer to cure (referral policy, policy for further diagnostics set?, known centre of expertise?, enough capacity in hospitals?) - Consequences for the primary process of the heel prick screening? - Organization of the screening in order? - Changes in quality policy required? - Adaptation of materials for communication and information needed? - Information- and communicationtechnology in order? - Monitoring and evaluation possible? - Ready for implementation? - Effect on costs? |

***Conditions***

| 1. Is the target disease for the screening defined without ambiguity? What are the envisaged findings? |  |
| --- | --- |
| 1. Is information available (within reasonable margins) on the prevalence of the condition in the Netherlands or internationally? |  |
| 1. Are there secondary findings that might be detected after following the various test steps? What secondary findings might be detected? Is information available for all possible incidental findings regarding prevalence, clinical relevance and the extent to which they are treatable? Is information available on how detection of these secondary findings may affect the benefit-risk ratio of screening? Is this effect acceptable? Can the secondary findings be shielded? If not, should the secondary findings be reported? If so, is information available on how that will take place? |  |
| 1. Will the screening detect mild variants of the condition? Is information available for these mild variants regarding prevalence, clinical relevance and the extent to which they are treatable? Is information available on how detection of these mild variants may affect the benefit-risk ratio of screening? Is this effect acceptable? |  |
| 1. Will the screening also identify genetic carriers of the condition? Is information available on what that carrier status means for those involved, in a physical and psychological sense? Is information available on how identification of these carriers may affect the benefit-risk ratio of screening? Is this effect acceptable? Do the genetic carriers have to be reported? If so, is information available on how that will take place? |  |

***Testing method***

| 1. Is information available on the different test steps? |  |
| --- | --- |
| 1. Can the different test steps be performed on the available dried   blood spot samples? |  |
| 1. Is information available on how much blood/sample material is needed for the different test steps? Is this amount of blood/sample material available? |  |
| 1. Do the different test steps place any exceptional requirements on storage of the blood/sample material? Can these requirements be met? |  |
| 1. Have the analytes for testing and the corresponding cut-off points (including those of any ratios or algorithms to be used) for the various test steps been determined, including for exceptional groups? |  |
| 1. Are post-analytical tools needed to achieve acceptable (clinical) sensitivity and specificity? If so, is the use of these post-analytical tools possible (from the start of screening)? |  |
| 1. Is information available on which equipment and test kits are needed to carry out the various test steps? If so, are these devices and test kits available? |  |
| 1. Are commercial tests of sufficient quality available for the various test steps, and do they offer sufficient guarantees for continuity of supply? If not, are other suitable tests available? |  |
| 1. Are the characteristics of the tests (analytical precision, accuracy, sensitivity and specificity) compliant with current quality standards? |  |
| 1. Do one or more test steps interfere with testing for other conditions? If so, is this acceptable or can it be changed in good time? |  |
| 1. Is information available on the requirements imposed on the laboratory set-up for implementation of the different test steps? Can these requirements be met at the start of screening? |  |
| 1. Is information available on what implementation of the different test steps will require in terms of laboratory logistics and personnel? Can the laboratories meet these requirements at the start of screening? |  |

***Predictive value***

| 1. What numbers of TP, FP and FN results are produced by the successive test steps? Are the numbers of FP and FN results after following the various test steps considered acceptable?   *Provisional estimate (per year):*   \| *TP – True positive* \|  \| \| --- \| --- \| \| *FP – False positive* \|  \| \| *FN – False negative* \|  \| |  |
| --- | --- | --- | --- | --- | --- | --- | --- |

***Timing of blood sampling***

| 1. Do the different test steps place any exceptional requirements on the timing of the neonatal blood spot screening? Can these specific requirements be met? If not, are the consequences for the benefit-risk ratio of screening considered acceptable? |  |
| --- | --- |

***Healthcare and transfer to cure***

| 1. Has the referral policy including referral terms been established, also for the Dutch Caribbean? Are those involved aware of this policy? |  |
| --- | --- |
| 1. Has the policy for further diagnostics and treatment been established, also for the Dutch Caribbean? Are those involved aware of this policy? |  |
| 1. Is it clear which centre of expertise is involved in diagnostics and treatment? What is the role of this centre of expertise? Are those involved aware of this? |  |
| 1. Is it clear how the screening will impact healthcare capacity (in terms of scope, expertise and funding)? Is this capacity available at the time that screening will start, or can it be made available? |  |

***Primary process***

| 1. Does the expansion of the neonatal heel prick screening to include this condition affect the primary process of the heel prick screening programme? |  |
| --- | --- |
| 1. Is an in-house test being used? If so, can all additional measures be taken in time for the condition to be added? |  |
| 1. Is sex selection applied during screening? If so, can all additional measures be taken in time for the condition to be added? |  |

***Organisation, tasks and responsibilities***

| 1. Is the organisation of the programme and of the various parties involved in the programme (particularly the Centre for Population Screening, the Department for Vaccine Supply and Prevention Programmes, screening laboratories, the reference laboratory, diagnostics and healthcare) sufficiently well-structured that the condition can be added to the neonatal heel prick screening programme? |  |
| --- | --- |

***Quality policy***

| 1. Does the scenario need to be adjusted? |  | |
| --- | --- | --- |
| 1. Is it necessary to adjust the national quality requirements (in the roadmap), and is it possible to do so in good time? |  | |
| 1. Is it necessary to adjust the guidance for healthcare providers (or arrange for it to be adjusted) and is it possible to do so in good time? |  | |
| 1. Does the expansion of the neonatal heel prick screening to include this condition have consequences for quality assurance? If so, can these consequences be mitigated? If so, by whom and how? |  | |
| 1. Is it possible (e.g. by organising national or regional meetings, adapting e-learning modules and providing instruction sessions for screeners) to promote expertise for the expansion of the neonatal heel prick screening to include this condition for the various groups of professionals within the given time frame? | |  |

***Information and communication***

| 1. Is it necessary and possible to arrange additional informed parental consent for the expansion of the neonatal heel prick screening to include this condition? |  |
| --- | --- |
| 1. Is it necessary and possible to adjust the general information materials for the expansion of the neonatal heel prick screening to include this condition? |  |
| 1. Is it necessary and possible to develop new results letters for the expansion of the neonatal heel prick screening to include this condition? |  |
| 1. Is it necessary and possible to adjust the Good Result Message for the expansion of the neonatal heel prick screening to include this condition? |  |
| 1. Is additional information needed for the RIVM Information Point and, if so, can this be achieved in time? |  |
| 1. Is additional information needed for the RIVM website (such as one or more FAQs) and, if so, can this be achieved in time? |  |

***Data management***

| 1. Will the new information system NHS (nwPraeventis) and the new LIMS be available in time to add this condition? If not, is it possible to adapt Praeventis and NEONAT in good time to support the newly added condition (or arrange for that to happen)? If not, are alternatives available with regard to data management? |  |
| --- | --- |
| 1. Is the ICT functionality flexible enough to handle the necessary process steps with regard to the heel prick card, add new letters to the system, set up short-cycle monitoring and log new indicators? |  |
| 1. Do the NEORAH and/or DDRMD database (registration database for metabolic diseases) need to be adapted? If so, can this been achieved in good time (including testing) before the start of the national screening? |  |

***Monitoring and evaluation***

| 1. Is it possible to subject the number of referrals and the number of repeated first heel pricks to short-cycle monitoring? |  |
| --- | --- |
| 1. Is it possible to closely monitor the results of diagnostics and the timely nature of diagnostics for this condition? |  |
| 1. Is it possible to develop new indicators for this condition to monitor the primary process properly? |  |
| 1. Is it possible to develop new target and signal values ​​for this condition? |  |
| 1. Is it necessary and possible to set up Long Term Follow Up for this condition? If so, what is the time frame and what would be needed for that? |  |

***Caribbean Netherlands***

| 1. Does the NHS-CN roadmap need to be adapted in terms of tasks and responsibilities for the new condition? If so, is it possible to achieve that adjustment? |  |
| --- | --- |
| 1. Is it necessary and possible to set up a pilot shipment to test whether the high temperatures and air humidity in the Caribbean Netherlands negatively affect the reliability of the analyses carried out in the screening laboratory in relation to the condition to be added? Will the results of that pilot be known in time for the condition to be added to the programme? |  |
| 1. Is it possible to arrange: a follow-up protocol for diagnostics and treatment of this condition, and approval in writing and confirmation by the BES Healthcare Office and by the Ministry of Health, Welfare and Sport arising from this follow-up protocol as a prescribed care path? |  |

***Implementation***

| 1. Does a tendering procedure still need to take place before the new condition can be added to the neonatal heel prick screening programme? If so, is this tendering procedure expected to be completed on time? |  |
| --- | --- |
| 1. Does anything need to be adapted in the legal regulations for the European or Caribbean Netherlands? If so, can this be achieved in time? |  |

***Costs***

| 1. Is it clear what the effect will be on the structural costs of adding the new condition, and is this effect included in the price of neonatal heel prick screening? |  |
| --- | --- |
